# Risk of Liver Fibrosis According to TSH Levels in Euthyroid Subjects

**DOI:** 10.3390/jcm10071350

**Published:** 2021-03-25

**Authors:** Alba Martínez-Escudé, Guillem Pera, Lluís Rodríguez, Ingrid Arteaga, Carmen Expósito-Martínez, Pere Torán-Monserrat, Llorenç Caballería

**Affiliations:** 1 Unitat de Suport a la Recerca (USR) Metropolitana Nord, Fundació Institut Universitari d’Investigació en Atenció Primària Jordi Gol i Gurina (IDIAP Jordi Gol), 08303 Mataró, Barcelona, Spain; gpera@idiapjgol.info (G.P.); lrodriguezg@gencat.cat (L.R.); iarteaga@gencat.cat (I.A.); cexposito.mn.ics@gencat.cat (C.E.-M.); ptoran.bnm.ics@gencat.cat (P.T.-M.); lcaballeria.bnm.ics@gencat.cat (L.C.); 2 Centre d’Atenció Primària La Llagosta, Institut Català de la Salut, 08120 La Llagosta, Barcelona, Spain; 3 Centro de Investigación Biomédica en Red de Enfermedades Hepáticas y Digestivas (CIBEREHD), 28029 Madrid, Spain; 4 Centre d’Atenció Primària Rocafonda-Palau, Institut Català de la Salut, 08303 Mataró, Barcelona, Spain; 5 Centre d’Atenció Primària Santa Eulàlia, Institut Català de la Salut, 08187 Santa Eulàlia de Ronçana, Barcelona, Spain; 6 Centre d’Atenció Primària Sabadell Centre, Institut Català de la Salut, 08201 Sabadell, Barcelona, Spain

**Keywords:** liver fibrosis, transient elastography, thyroid, thyroid function, thyrotropine, non-alcoholic fatty liver disease

## Abstract

Alterations in thyroid function may contribute to the development of liver fibrosis especially in subjects with non-alcoholic fatty liver disease. This study aimed to investigate the risk of liver fibrosis according to low-normal thyroid function in the general population. We performed a descriptive cross-sectional study in subjects from 18–75 years randomly selected from 16 primary health care centers from 2017–2019. Each subject underwent clinical evaluation, physical examination, blood analysis and transient hepatic elastography. Descriptive and multivariate logistic regression analyses were used to identify factors associated with fibrosis. We included 1096 subjects (60 ± 11 years; 61% women); 70% had strict-normal thyroid function and 30% had low-normal thyroid function. Low-normal thyroid function was associated with a higher liver stiffness (LS) values: 5.2 vs. 4.8 kPa (*p* = 0.001) and a greater prevalence of fibrosis: 6.1 vs. 3% (*p* = 0.016) and 4.3 vs. 2.1% (*p* = 0.044) for the cut-off points of ≥8.0 kPa and ≥9.2 kPa, respectively. After adjustment for potential confounding factors, the risk of fibrosis in subjects with low-normal thyroid function was OR 1.54 (*p* = 0.213). In conclusion, low-normal thyroid function is associated with higher LS values and a greater risk of liver fibrosis in the general population, being dependent on other metabolic factors.

## 1. Introduction

Liver fibrosis in subjects with chronic liver disease is a factor in bad prognosis for the development of liver cirrhosis and its consequent complications [1]. The prevalence of fibrosis in the general population ranges from 3.6 to 5.8% according to the diagnostic method used [2]. The main causes of liver fibrosis are alcohol intake, viral hepatopathies and non-alcoholic fatty liver disease (NAFLD). The latter is one of the most frequent liver diseases in our setting, affecting one fourth of the population with a prevalence that has shown to be exponentially increasing in the last years due to the rise in obesity, type 2 diabetes mellitus (T2DM) and the metabolic syndrome (MetS) [3]. At present, there are no antifibrotic treatments able to reverse or slow the progression of histological liver damage. It is essential to identify the risk factors associated with liver fibrosis in order to approach the disease from its initial or silent stages.

It has recently been suggested that thyroid hormones may influence the development of NAFLD and the progression of liver fibrosis [4]. In some studies, hypothyroidism has been associated with NALFD independently of other factors [5], however, this relationship was not found in a recently published study including a large number of subjects [6]. On the other hand, the association between low thyroid function and liver fibrosis has also been studied, although this is still controversial [7,8]. The pathogenic mechanisms are not well defined but some common factors such as insulin resistance (IR), oxidative stress or MetS may be involved.

On the other hand, it has been suggested that low-normal thyroid function, that is, high thyroid stimulating hormone (TSH) or lower thyroxine (T4) levels within the euthyroid range, could induce similar health effects similar to those observed in hypothyroid subjects [9]. In a recent study including patients with NAFLD, low thyroid function, defined as TSH ≥ 2.5 μIU/mL, was independently associated with the presence of steatohepatitis and advanced fibrosis (F3–F4) in liver biopsy [10].

Although the association between hypothyroidism, NAFLD and liver fibrosis has been studied, there are few studies on the impact of low-normal thyroid function within the euthyroid range on the pathogenesis of fibrosis. Therefore, the aim of the present study was to investigate the risk of liver fibrosis according to low-normal thyroid function in the general population.

## 2. Methods

### 2.1. Study Design and Population

This was a descriptive, cross-sectional, multicenter population-based study. The participants included subjects from 18 to 75 years from 16 primary health care centers from the area of Barcelonès Nord and Maresme (Catalonia, Spain).

These subjects were randomly selected from the Primary Care Information System (Spanish ancronym SIAP) which is a populational database equivalent to the census in Catalonia. The exclusion criteria for the initial sample selection were: previously diagnosed chronic liver diseases, advanced severe diseases, cognitive impairment, institutionalized patients and death.

The study population was obtained from the follow-up of the populational cohort of 3014 subjects included in the recently published study by our group on the detection of liver diseases in the general population carried out from 2012 to 2016 [2]. Of the total of 3014 subjects contacted, 1684 accepted to participate in the follow-up by telephone, representing 56% of the total. Each participant underwent a clinical interview, physical examination, blood analysis and transient elastography (TE). Data were collected from January 2017 to December 2019. For the analysis we excluded subjects with incomplete laboratory data (*n* = 367), hyperthyroidism or hypothyroidism (*n* = 79), absence of or invalid elastography measurements (*n* = 32) and alcohol risk intake (*n* = 110) defined by a weekly alcohol intake ≥21 standard drink units (SDU) in men and ≥14 SDU in women. The final sample included was 1096 subjects.

The study protocol was approved by the IDIAP Jordi Gol Ethical Committee (P14/123) and was performed following the norms of the Declaration of Helsinki. All the subjects provided signed informed consent prior to inclusion, and the data were managed according to state legislation on data protection (LOPDGDD 3/2018).

### 2.2. Clinical and Laboratory Parameters

The following variables were collected: sociodemographic data: age and sex; anthropometric data: height, weight, waist circumference (WC) and the body mass index (BMI: weight in kg/height in m^2^); systolic (SBP) and diastolic blood pressure (DBP); consumption of toxic substances: tobacco and alcohol in SDU; presence of comorbidities: arterial hypertension (AHT), hypercholesterolemia, hypertriglyceridemia, overweight and obesity, T2DM, MetS and NAFLD.

Blood analyses were performed after 12h of fasting and included the determination of: complete blood count, glycemia, glycosylated hemoglobin, total cholesterol, high-density lipoproteins (HDL), low-density lipoproteins (LDL), triglycerides (TG); TSH, T4; alanine aminotransferase (ALT), aspartate aminotransferase (AST), gamma glutamyltransferase (GGT), alkaline phosphotase (ALP); total proteins and albumin.

### 2.3. Definitions

Euthyroidism was defined as TSH values between 0.35–4.94 μIU/mL and T4 1.7–1.48 μIU/mL, according to data from our reference laboratory. The subjects were classified into two groups for comparison: those presenting strict-normal thyroid function (TSH ≥ 0.35 μIU/mL and <2.5 μIU/mL; with normal T4 values) and those presenting low-normal thyroid function (TSH ≥ 2.5 μIU/mL and ≤4.94 μIU/mL; with normal T4 values).

MetS was diagnosed according to the criteria of the National Cholesterol Education Program—Adult Treatment Panel III (NCEP-ATPIII) [11], when the subjects presented 3 or more of its components: WC > 88 cm in women and > 102 cm in men; TG ≥ 150 mg/dl or on hypolipemiant treatment; HDL < 40 mg/dL in men and < 50 mg/dL in women or on hypolipemiant treatment; blood pressure ≥ 130/85 mmHg or on hypotensive treatment; and basal glycemia ≥ 100 mg/dL or on hypolglycemia treatment.

The diagnosis of NAFLD was made using the fatty liver index (FLI) serological marker, according to B2 recommendations of the European guidelines [12]. The FLI includes the variables of TG, BMI, GGT and WC and is calculated based on the following formula:

FLI = (e^0.953 × loge(TG) + 0.139 × BMI + 0.718 × loge(GGT) + 0.053 × WC−15.745^)/(1 + e^0.953 × loge(TG) + 0.139 × BMI + 0.718 × loge(GGT) + 0.053 × WC−15.745^) × 100 (1)

A FLI score ≥ 60 is diagnostic of NAFLD, while a FLI score of 30–60 is indeterminate, and a score < 30 indicates no NAFLD.

### 2.4. Evaluation of Liver Fibrosis

#### 2.4.1. Transient Elastography (TE)

This was performed by a previously trained nurse using the Fibrosan 402 device (Echosens, París, France) equipped with an M probe, in all the study subjects. Subjects lacking 10 valid measurements and/or an interquartile range (IQR) of the measurement greater than 30% were excluded. Two cut-off points were established for the diagnosis of fibrosis according to the values of liver stiffness (LS) of ≥8.0 kilopascals (kPa) and ≥9.2 kPa (suggestive of significant liver fibrosis ≥F2) [13].

#### 2.4.2. Serological Markers

NAFLD fibrosis score (NFS): this score includes the variables of age, BMI, altered basal glycemia (ABG), AST, ALT, platelets and albumin and is calculated according to the following formula:

NFS = −1.675 + (0.037 × age) + (0.094 × BMI) + (1.13 × ABG/diabetes) + (0.99 × AST/ALT ratio) − (0.013 × platelets [× 10^9^/L]) − (0.66 × albumin [g/dL]) (2)

FIB-4: includes age, AST, ALT and platelets in the formula:

FIB-4 = (age × AST)/(Platelets × √(ALT))(3)

The aspartate aminotransferase to platelet ratio index (APRI): includes AST, the upper limit of normality for AST and platelets using the formula:

APRI = (AST in IU/L)/(AST upper limit of normality in U/L)/(Platelets in 10^9^/L) (4)

The criteria for predicting liver fibrosis according to the serological markers [13,14] were: NFS > 0.675; FIB-4 > 3.25 and APRI > 1.5.

### 2.5. Statistical Analysis

Continuous variables are expressed as means and standard deviation, except for those that do not have a normal distribution, which are presented as medians and IQR. Categorical variables are expressed as frequencies and percentages. The prevalences were calculated with their respective 95% confidence intervals (95% CI).

For the comparison of variables, two groups were established based on thyroid function: strict-normal and low-normal. The chi-square test was used for categorical variables, while the Student’s t test was used for continuous variables with a normal distribution and the Mann-Whitney test for variables expressed as medians.

The outcome variable was the presence of liver fibrosis defined by the LS values using two alternative cut-off points in the TE: ≥8.0 kPa and ≥9.2 kPa. In addition, an analytical criterion was used to define liver fibrosis, which was the presence of at least one altered serological marker (NFS, FIB-4 and APRI). To evaluate whether low-normal thyroid function was independently associated with liver fibrosis, bivariate and multivariate logistic regression analyses were used adjusted for potential confounding factors. The corresponding odds ratios (OR) and their 95% CI were obtained.

All the statistical tests were performed with bilateral contrasts considering statistical significance with a *p* value < 0.05. The analyses were carried out with the Stata versión 15 package (Stata-Corp, College Station, TX, USA).

## 3. Results 

### 3.1. Basal Characteristics

Of the 1096 subjets included (mean age 60 ± 11 years; 61% women), 767 (70%) were classified in the group of strict-normal thyroid function, and 329 (30%) were included in the low-normal thyroid function group. Table 1 shows the basal characteristics of the study subjects. It was of note that among the participants with low-normal thyroid function, there was a greater prevalence of global obesity (38%; *p* = 0.001), abdominal obesity (57%; *p* = 0.002) and MetS (34%; *p* < 0.001) compared to those with strict-normal thyroid function. Physical examination showed a greater BMI (*p* < 0.001) and higher WC in women with low-normal thyroid function (*p* = 0.005), as well as higher TG values (125 ± 59 vs. 111 ± 54 mg/dL; *p* < 0.001) in blood analyses.

The global prevalence of NAFLD was 37% (*n* = 402). Differences were found in regard to the prevalence of NAFLD according to thyroid function, being 44% in subjects with low-normal thyroid function (*p* = 0.002). Hypertransaminasemia, defined as AST and/or ALT >35 U/L, affected 10% of the study subjects. The low-normal thyroid function group showed higher ALT values (23 ± 16 vs. 21 ± 12 U/L; *p* = 0.034). Although the prevalence of hypertransaminasemia was greater in the low-normal thyroid function group (12% vs. 9.5%), these results were not significant.

### 3.2. Prevalence of Fibrosis According to Thyroid Function

Elastrography values were ≥8.0 kPa and ≥9.2 kPa in 3.92% and 2.7% of the subjects, respectively. Subjects with low-normal thyroid function had higher LS values compared to those with strict-normal thyroid function (5.2 vs. 4.8 kPa; *p* = 0.001). The prevalence of fibrosis was also greater in the group with low-normal thyroid function compared to the control group being 6.1% vs. 3% (*p* = 0.016) with ≥8.0 kPa and 4.3% vs. 2.1% (*p* = 0.044) with ≥9.2 kPa. To the contrary, there were no statistically significant differences in the prevalence of liver fibrosis based on serological markers and thyroid function (Table 2).

### 3.3. Relationship between Thyroid Hormones and Liver Fibrosis

On the other hand, higher TSH values were found in subjects with significant fibrosis by TE in both cut-off points for LS 8.0 kPa and 9.2 kPa (Table 3). There were no differences in T4 values.

**Figure 1:**
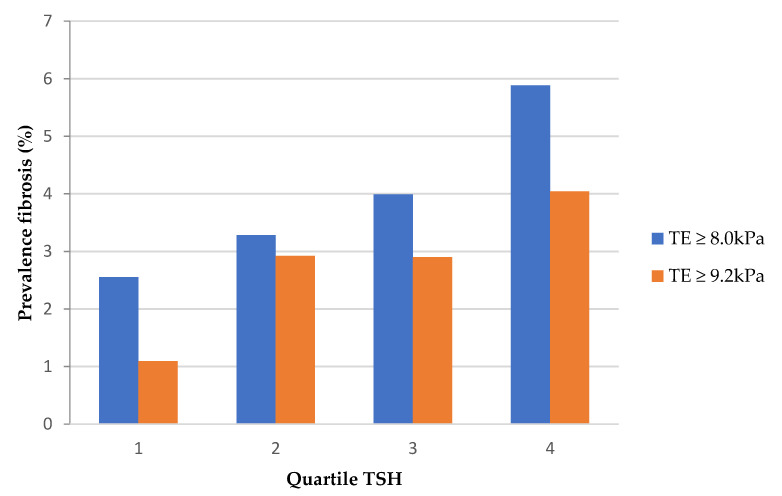
Prevalence of liver fibrosis according to TSH quartiles. **Abbr:** TE, transient elastography; TSH, thyroid stimulating hormone.

The prevalence of fibrosis showed a dose-dependent increase with an increase in TSH values (Figure 1).

An excess of risk of liver fibrosis of 1.44 was observed for each increase in TSH levels, independently of age, sex or alcohol intake. This excess risk was also independent of obesity and MetS for LS values ≥ 8.0 kPa (Table 4).

### 3.4. Risk of Fibrosis According to Thyroid Function

The univariate analysis detected an increase in the risk of fibrosis in subjects with low-normal thyroid function which was 2.09-fold greater for both LS values ≥ 8.0 kPa and ≥9.2 kPa (Table 5).

Multivariate analysis adjusted for age, sex and alcohol intake showed a significant association between low-normal thyroid function and elastrography values of ≥8.0 kPa (OR 2.11) and ≥9.2 kPa (OR 2.08). To evaluate whether the association between low-normal thyroid function and fibrosis by elastography was independent of other factors, multivariate analyses were performed using separate models, adjusted for obesity, MetS and NAFLD. We observed no independent or significant increase in the risk of fibrosis in subjects with low-normal thyroid function for the two elastography cut-off points used in relation to the presence of obesity, MetS or NAFLD (Table 6).

On the other hand, in an analysis adjusted for the specific components of MetS (Table 7), both WC and glycemia were significantly associated with fibrosis with the LS values ≥ 8.0 kPa and ≥9.2 kPa. TG were related to LS values ≥ 9.2 kPa and HDL with LS ≥ 8.0 kPa, while BP was not associated with liver fibrosis. To the contrary, low-normal thyroid function was not found to be an independent factor of the MetS components for fibrosis with LS values ≥ 8.0 kPa (OR 1.54; *p* = 0.213) and ≥9.2 kPa (OR 1.42; *p* = 0.391).

Finally, neither was any association found between low-normal thyroid function and the risk of fibrosis using the serological markers analyzed in the multivariate analysis adjusted for age, sex, alcohol intake and the different parameters of MetS (OR 0.84; 95%CI 0.63–1.13; *p* = 0.244).

## 4. Discussion

The findings of the present study demonstrate that low-normal thyroid function is associated with a two-fold greater risk of liver fibrosis compared to strict-normal thyroid function. To our knowledge, this is the first European study to evalulate the risk of liver fibrosis in the general population according to thyroid function within the euthyroid range. Although these results are clinically relevant, the increase in the risk found was not independent of parameters of the MetS or the other factors studied.

It is well known that thyroid hormones participate in multiple processes of metabolism, such as lipolysis, neoglucogenesis as well as the regulation of weight and temperature. The effects of low thyroid function on health, specifically hypothyroidism, include a greater prevalence of obesity, dyslipemia, MetS and greater IR, which are determinant factors for the development of NAFLD [15,16,17]. At a hepatic level, thyroid hormones are involved in beta oxidation of the fatty acids and could influence the accumulation of fat in the liver. The main thyroid hormone receptor (THR) expressed in the liver is THRβ and its role was demonstrated in a study designed with mice, where a dominant negative mutation in THRβ was analyzed and it was observed that these mice developed hepatic steatosis in a few months [18]. Other physiopathological mechanisms involved in NAFLD/NASH such as the role of adipocytokines, oxidative stress reactions, mitochondrial dysfunction or lipid peroxidation have also been related to thyroid hormones [19]. The activation of hepatic stellate cells is an important step in liver fibrogenesis [20]. In case of liver injury, it has been suggested that inhibition of nuclear THR expression may activate hepatic stellate cells favoring the fibrogenic response [19,21].

Some studies have demonstrated results similar to those of our study, although few studies have evaluated the effect of low-normal thyroid function within the euthyroid range on liver fibrosis. Kim et al. [10] demonstrated that subjects with NAFLD and low thyroid function, defined as TSH ≥ 2.5 μIU/mL, have a greater risk of developing non-alcoholic steatohepatitis (NASH) and advanced fibrosis (stages F3–F4 in liver biopsy). Another study carried out in the general population, including 7259 participants, found an increase in the risk of advanced fibrosis defined by serological markers, which was 2-fold higher in subjects with low-normal thyroid function with respect to a group with strict-normal function independently of the WC, cholesterol values or IR [22]. To the contrary, in our study there was no association between low-normal thyroid function and an alteration in serological markers of fibrosis.

The effects of thyroid hormones on liver fibrosis have also been studied. Specifically, TSH levels have been associated with LS values ≥ 8.0 kPa in patients with NAFLD diagnosed by FLI [23]. We also found an increase in TSH values in euthyroid subjects with liver fibrosis as well as an excess of risk of LS ≥ 8.0 kPa, of 1.44 for each increase in TSH unit independently of the presence of obesity or MetS. On the other hand, Manka et al. found an association between low T3 levels and liver fibrosis (by TE or serological markers) but could not demonstrate the relationship between TSH and T4 levels with LS in subjects with NASH [8].

One aspect of note in the present study, which excluded the main causes of chronic liver disease including alcohol risk intake, was that the prevalence of fibrosis found might be attributed to NAFLD in most of the cases. In fact, the prevalence of NAFLD in subjects with low-normal thyroid function was significantly greater, affecting almost half of these individuals.

Other authors have studied the role of thyroid hormones within the reference range in NAFLD. Low T4 levels have been associated with the risk of NAFLD in euthyroid subjects [24], and in some studies this association was independent of the presence of MetS [25]. On the other hand, high TSH levels have been related to NAFLD [26,27]. In a recent meta-analysis including 61,548 subjects, there was a significant increase in TSH values in subjects with NAFLD compared to a control group, with a weighted mean difference of 0.105 (95%CI 0.012–0.197), concluding that this could be a risk factor for the development and progression of NAFLD [28]. In contrast, this association has not been demonstrated in other studies [29].

According to the data available, low-normal thyroid function has been related to moderate increases in total cholesterol, LDL and TG values [30]. Likewise, it has been linked to greater IR and an increase in the risk of MetS similar to what occurs in patients with hypothyroidism [31,32]. Elevated TSH levels, even within the reference range, have also been related to an increase in central obesity, among other alterations such as hyperglycemia, hyperuricemia, elevation in blood pressure, hypercoagulability or an increase in inflammatory markers [33]. Thus, our study shows similar results with increases in the prevalence of obesity and MetS and elevations in TG levels in subjects with low-normal thyroid function. These findings are clinically important, since these factors may not only have implications in the cardiovascular system but may exacerbate the development of NAFLD and liver fibrosis given the common physiopathogenic mechanisms. In fact, in a study with a mean follow-up of 23 years, the univariate model demonstrated that low thyroid function (including low-normal euthyroidism and hypothyroidism) was associated with a greater risk of mortality in subjects with NAFLD [34]. Taking all of this into account, some authors have suggested that the reference range of normality for thyroid function should be reevaluated.

The present study has some limitations. The cross-sectional design did not allow the determination of a relation of causality among the variations of thyroid function and liver fibrosis. The gold standard method for determining the grade of fibrosis is liver biopsy [35], but since this is an invasive test it cannot be routinely performed. In our case, we used validated serological markers and measurements of LS by TE, which has a sensitivity of 95–98% [36]. The XL probe is recommended for measuring LS in subjects with obesity, but in our study all the TE were performed with the M probe because it was the only probe available. Finally, thyroid hormones values may have undergone temporal modifications and the impact of this on the liver is unknown.

## 5. Conclusions

In conclusion, the findings of this study demonstrate that low-normal thyroid function is associated with a greater risk of liver fibrosis in the general population, although dependently of other metabolic factors. These data demonstrate the need to reconsider the objectives of thyroid hormones control in patients with risk factors for the development of liver fibrosis.

## Figures and Tables

**Table 1:** Basal characteristics of the subjects according to thyroid function (*n* = 1096).

	Strict-Normal Thyroid Function (*n* = 767)	Low-Normal Thyroid Function (*n* = 329)	*p* Value
Age (years)	60 ± 11	61 ± 11	0.020
Female	437 (57%)	229 (70%)	<0.001
Toxic substances			
Smoking Ever	425 (55%)	159 (49%)	0.035
Alcohol (SDU/week) *	0 ± 4	0 ± 2	0.001
Disease history			
T2DM	104 (14%)	55 (17%)	0.174
HBP	276 (36%)	125 (38%)	0.527
Hypercholesterolemia	296 (39%)	144 (44%)	0.109
Hypertriglyceridemia	85 (11%)	50 (15%)	0.057
Global obesity			0.001
*Normal weight*	197 (26%)	75 (23%)	
*Overweight (BMI ≥ 25 to <30)*	360 (47%)	128 (39%)	
*Obesity (BMI ≥ 30)*	210 (27%)	126 (38%)	
Abdominal obesity			
*Overall*	353 (46%)	185 (57%)	0.002
*Male*	98 (30%)	36 (36%)	0.223
*Female*	255 (59%)	149 (65%)	0.092
MetS	184 (24%)	113 (34%)	<0.001
FLI ≥ 60	259 (34%)	143 (44%)	0.002
Physical examination			
BMI	28 ± 4	29 ± 5	<0.001
WC-Male (cm)	98 ± 10	100 ± 11	0.106
WC-Female (cm)	91 ± 12	94 ± 13	0.005
SBP (mmHg)	125 ± 17	125 ± 17	0.568
DBP (mmHg)	79 ± 10	79 ± 9	0.719
Blood analysis			
Glycemia (mg/dL)	100 ± 24	101 ± 24	0.669
Glycosylated hemoglobin (%)	5.7 ± 0.8	5.7 ± 0.7	0.964
Total cholesterol (mg/dL)	207 ± 37	208 ± 39	0.625
HDL (mg/dL)	55 ± 13	54 ± 13	0.174
LDL (mg/dL)	130 ± 33	130 ± 38	0.854
TG (mg/dL)	111 ± 54	125 ± 59	<0.001
TSH (μIU/mL)	1.6 ± 0.5	3.3 ± 0.6	<0.001
T4 (μIU/mL)	0.98 ± 0.1	0.95 ± 0.1	<0.001
ALT (U/L)	21 ± 12	23 ± 16	0.034
AST (U/L)	22 ± 8	23 ± 9	0.220
ALT and/or AST > 35 U/L	73 (9.5%)	40 (12%)	0.188
GGT (U/L)	30 ± 29	30 ± 35	0.933
ALP (U/L)	79 ± 22	81 ± 23	0.284
Platelets (10^9^/L)	223 ± 55	228 ± 58	0.162

All results are expressed in frequency (%) or mean ± standard deviation, except for those with * that are expressed in median ± interquartil range. Abbr: T2DM, type 2-diabetis mellitus; HBP, high blood pressure; MetS, metabolic syndrome; FLI, fatty liver index; BMI, body max index; WC, waist circumference; SBP, systolic blood pressure; DBP, diastolic blood pressure; HDL, high density lipoprotein; LDL, low density lipoprotein; TG, triglycerides; TSH, thyroid stimulating hormone; T4, thyroxine; ALT, alanine aminotransferase; AST, aspartate aminotransferase; GGT, g-glutamyltransferase; ALP, alkaline phosphatase.

**Table 2:** Association between liver fibrosis according to thyroid function.

	Strict-Normal Thyroid Function (*n* = 767)	Low-Normal Thyroid Function (*n* = 329)	*p* Value
Transient elastography			
kPa ± SD	4.8 ± 1.6	5.2 ± 3.0	0.001
≥8.0 kPa	23 (3.0%)	20 (6.1%)	0.016
≥9.2 kPa	16 (2.1%)	14 (4.3%)	0.044
Serologic markers			
NFS > 0.675	38 (5.0%)	24 (7.4%)	0.120
FIB4 > 3.25	8 (1.1%)	8 (2.5%)	0.078
APRI > 1.5	0 (0%)	2 (0.6%)	0.031

All results are expressed in frequency (%) or mean ± standard deviation.

**Table 3:** Association between thyroid hormones and liver fibrosis using transient elastography.

	Transient Elastography (TE)					
	TE < 8.0 kPa	TE ≥ 8.0 kPa	*p* Value	TE < 9.2 kPa	TE ≥ 9.2 kPa	*p* Value
TSH (μIU/mL)	2.1 ± 1.0	2.4 ± 1.0	0.015	2.1 ± 1.0	2.5 ± 1.0	0.034
T4 (μIU/mL)	0.97 ± 0.10	0.95 ± 0.11	0.130	0.97 ± 0.10	0.96 ± 0.13	0.546

All results are expressed in mean ± standard deviation.

**Table 4:** Analysis between TSH levels (μIU/mL) and risk of liver fibrosis, using different transient elastography cut-offs as dependent variables. Logistic regression models.

	TE ≥ 8.0 kPa	TE ≥ 9.2 kPa
	OR (95%CI) *p* value	OR (95%CI) *p* value
Univariate	1.43 (1.07–1.92) 0.016	1.45 (1.02–2.05) 0.036
Multivariate *	1.44 (1.07–1.93) 0.016	1.44 (1.02–2.05) 0.039
Adjusted for BMI ≥ 30	1.37 (1.02–1.86) 0.039	1.37 (0.96–1.95) 0.086
Adjusted for MetS	1.33 (0.99–1.80) 0.059	1.33 (0.93–1.88) 0.115
Adjusted for FLI ≥ 60	1.28 (0.95–1.74) 0.106	1.26 (0.88–1.81) 0.201

* All multivariate analyses are adjusted also for age, sex and alcohol intake. Correlation between TSH levels and TE (kPa) r = 0.07. Abbr: BMI, body max index; MetS, metabolic syndrome; FLI, fatty liver index; TE, transient elastrography; OR, odds ratio; CI, confidence interval; TSH, thyroid stimulating hormone.

**Table 5:** Analysis of risk of liver fibrosis using different transient elastography cut-offs as dependent variables, in low-normal thyroid function (TSH 2.5–4.94 μIU/mL) vs. strict-normal thyroid function (TSH < 2.5 μIU/mL). Logistic regression models.

	TE ≥ 8.0 kPa	TE ≥ 9.2 kPa
	OR (95%CI) *p* value	OR (95%CI) *p* value
Univariate	2.09 (1.13–3.87) 0.018	2.09 (1.01–4.33) 0.048
Multivariate *	2.11 (1.13–3.95) 0.019	2.08 (0.99–4.36) 0.053

* Also adjusted for age, sex and alcohol intake. Abbr: TE, transient elastography; OR, odds ratio; CI, confidence interval; TSH, thyroid stimulating hormone.

**Table 6:** Multivariate analysis of risk of liver fibrosis using different transient elastography cut-offs as dependent variables in low-normal thyroid function (TSH 2.5–4.94 μIU/mL) vs. strict-normal thyroid function (TSH < 2.5 μIU/mL). Three different multivariate logistic regression models adjusted for BMI, MetS and FLI.

	TE ≥ 8.0 kPa	TE ≥ 9.2 kPa
	OR (95%CI) *p* value	OR (95%CI) *p* value
TSH 2.5–4.94 (μIU/mL) *	1.78 (0.94–3.36) 0.077	1.68 (0.79–3.56) 0.180
BMI ≥ 30	6.63 (3.27–13.46) < 0.001	11.31 (4.25–30.06) < 0.001
TSH 2.5–4.94 (μIU/mL) *	1.78 (0.94–3.37) 0.075	1.71 (0.80–3.64) 0.163
MetS	5.58 (2.81–11.08) < 0.001	7.35 (3.12–17.34) < 0.001
TSH 2.5–4.94 (μIU/mL) *	1.66 (0.87–3.16) 0.123	1.58 (0.73–3.38) 0.244
FLI ≥ 60	10.21 (4.21–24.73) < 0.001	10.45 (3.56–30.65) < 0.001

* All multivariate analyses are also adjusted for age, sex and alcohol intake. Each model shows the OR for TSH (in bold) and the specific adjusted variable (BMI, MetS, FLI) ORs. No interaction between low-normal thyroid function, obesity, MetS and NAFLD in the statistical analysis (*p* value ≥ 0.2). Abbr: BMI, body max index; MetS, metabolic syndrome; FLI, fatty liver index; TSH, thyroid stimulating hormone; TE, transient elastrography.

**Table 7:** Multivariate analysis of risk of liver fibrosis using different transient elastography cut-offs as dependent variables in low-normal thyroid function (TSH 2.5–4.94 μIU/mL) vs. strict-normal thyroid function (TSH < 2.5 μIU/mL). Multivariate logistic regression models adjusted for all the different parameters of MetS.

	TE ≥ 8.0 kPa	TE ≥ 9.2 kPa
	OR (95%CI) *p* value	OR (95%CI) *p* value
TSH 2.5–4.94 (μIU/mL) *	1.54 (0.78–3.02) 0.213	1.42 (0.64–3.13) 0.391
WC > 88/ > 102 cm female/male	6.90 (2.53–18.78) < 0.001	7.33 (2.08–25.86) 0.002
TG ≥ 150 mg/dL	1.99 (0.97–4.08) 0.060	2.58 (1.11–6.01) 0.028
HDL < 50/40 mg/dL female/male	2.30 (1.11–4.76) 0.025	2.27 (0.95–5.40) 0.063
BP ≥ 130/85 mmHg	0.57 (0.29–1.13) 0.110	0.69 (0.31–1.52) 0.355
Glucose ≥ 100 mg/dL	3.95 (1.76–8.89) < 0.001	3.38 (1.30–8.79) 0.013

* Also adjusted for age, sex and alcohol intake. All variables mutually adjusted. Abbr: WC, waist circumference; TG, trygliceride; HDL high-density lipoprotein; BP, blood pressure; TSH, thyroid stimulating hormone; TE, transient elastrography.

## Data Availability

Data sharing not applicable.
